# Ancient anomalies: Twinned and supernumerary incisors in a medieval Nubian

**DOI:** 10.1002/oa.2954

**Published:** 2021-01-26

**Authors:** Emma L.W. Phillips, Joel D. Irish, Daniel Antoine

**Affiliations:** ^1^ School of Biological and Environmental Sciences Liverpool John Moores University Liverpool UK; ^2^ Department of Egypt and Sudan The British Museum London UK

**Keywords:** ancient Sudan, dental anomalies, fusion, gemination, supernumerary tooth, twinned crown

## Abstract

During the analysis of a skeletal assemblage from a medieval cemetery in Nubia (c. AD 500–1550), a young adult female with abnormally developed maxillary incisors was discovered. The possible causes of the two dental anomalies found in this individual and their archaeological context are discussed. The remains are from a medieval assemblage from the Fourth Cataract region of Nubia, which forms part of the Nubian collection curated at the British Museum. The left central incisor has a twinned crown with two root canals, and a supernumerary tooth is present on the right side between the central incisor and lateral incisors. Although two different dental anomalies are present, the bilateral expression suggests that the same biological mechanism could be responsible.

## 1 INTRODUCTION

This report describes two different dental anomalies observed in the teeth of a young skeletonized female dating to the medieval period of Upper Nubia (i.e., present‐day northern Sudan). Her upper left central incisor had developed abnormally, with two root canals and a twinned crown. In addition, a supernumerary tooth had formed on the right maxilla between the central and lateral incisors. Both conditions have been detailed in the clinical literature but are rarely documented in archaeological contexts. This case offers an opportunity to discuss the potential etiologies and expression of such anomalies from a deep time perspective, highlighting that dental developmental issues are not unique to modern peoples.

Dental development is a stable and evolutionarily conserved process. The formation of teeth involves interactions between networks of activators and inhibitors under tight genetic control (Bei, 2009). This process appears to have been the same for millions of years, showing little change (Scott et al., 2018). Any alteration can result in abnormalities in the form or patterning of teeth (Hlusko, 2016). Although understanding of how genetic, epigenetic, and environmental influences interplay during odontogenesis has been greatly advanced (Townsend et al., 2012), the etiology of these abnormalities remains unclear.

There are two main processes that result in a double crown: gemination and fusion. The first occurs when a tooth bud fails to divide (Koszowski et al., 2014). This partial division results in a twinned crown and usually a common root and root canal, though separate root canals have also been known to occur (Mahendra et al., 2014). Sometimes a notch forms in the incisal edge, where the two crowns have attempted to separate (Mahendra et al., 2014). A gemmate tooth does not add to tooth count (Koszowski et al., 2014). Fusion can also produce teeth with double crowns, with the union of two adjacent tooth buds during development (Benazzi et al., 2010). This probably occurs early in development, when the crowns have yet to be mineralized (Koszowski et al., 2014). Most clinical literature agrees that there has to be a union of the dentine, and the process can result in a shared pulp chamber and root canal, or both elements can remain separate (Benazzi et al., 2010; Koszowski et al., 2014). Unless it involves a supernumerary tooth, fusion often results in a reduction of tooth number in the dental arch (Garattini et al., 1999; Nunes et al., 2002).

Gemmate teeth are more common in the anterior maxillary arch (Koszowski et al., 2014), whereas fused teeth most frequently affect mandibular incisors (Benazzi et al., 2010). Though the two processes occur in both sets of dentitions, incidences are lower in permanent teeth (Benazzi et al., 2010; Koszowski et al., 2014). Population differences have also been noted, but anomalies are usually found in around 0.1–0.2% of permanent dentitions (Koszowski et al., 2014; Mahendra et al., 2014). Because both gemination and fusion can create teeth that appear morphologically and physiologically similar, it can be difficult to diagnose correctly the mechanism involved, necessitating a reliance on tooth count (Mahendra et al., 2014). The situation can be even more complex to interpret when fusion involves a supernumerary tooth (Benazzi et al., 2010). Several etiologies have been proposed for gemination and fusion including evolution, systemic diseases, hypervitaminosis A, trauma, pressure, heredity, and environmental influences (Benazzi et al., 2010; Koszowski et al., 2014; Mahendra et al., 2014).

Teeth that develop in addition to the 32 permanent and 20 deciduous teeth are termed ‘supernumerary’ (Anthonappa et al., 2013; Takahashi et al., 2016). They can be heteromorphic in shape (conical, tuberculate or odontome) or eumorphic (aka supplemental) (Garvey et al., 1999; Rajab & Hamdan, 2002), and have been observed at varying levels of mineralization and development (Takahashi et al., 2016). Supernumerary teeth can occur anywhere in both dental arches but are most often present in the incisor or molar regions (Anthonappa et al., 2013; Bailleul‐Forestier et al., 2008; Rajab & Hamdan, 2002). The incidence can be unilateral (76–78%) or bilateral (12–23%) (Takahashi et al., 2016). The presence of these teeth, especially more than two, have also been linked to several genetic syndromes (e.g., Gardener's Syndrome; Anthonappa et al., 2013). Many researchers have also observed that supernumerary teeth are more prevalent in males (Takahashi et al., 2016). The presence of supernumerary teeth varies between populations, at both global and local levels. Their prevalence is thought to range between 0.1% and 3.8% (Bailleul‐Forestier et al., 2008; Takahashi et al., 2016).

Multiple theories have been proposed to explain the occurrence of supernumerary teeth (Takahashi et al., 2016). Although most researchers agree on the hereditary nature of supernumerary teeth, there is no consensus as to why these teeth occur or what mechanisms are involved (Anthonappa et al., 2013; Bailleul‐forestier et al., 2008; Takahashi et al., 2016). In recent years, the idea of independent and localized dental lamina hyperactivity has emerged as the most likely cause (Duncan, 2009). Dental lamina is the thickening of the oral epithelium, starting the process of tooth development in the uterus (Wang & Fan, 2011). Reactivation of the dental lamina, induced by signaling molecules (including WNTs and BMPs, bone morphogenetic proteins), initiates the formation of secondary dentition (Järvinen et al., 2009). Once crowns of the permanent dentition have been created, signaling molecules activate dental lamina apoptosis and degeneration (Wang & Fan, 2011). Interruptions to the complex signaling pathways can cause prolonged survival or overproliferation of the lamina, resulting in supernumerary teeth (Tummers & Thesleff, 2009).

Other research has highlighted the role dental lamina plays in dichotomy. Dichotomy occurs when the dental lamina separates into two parts. These portions develop and produce two separate tooth germs (Wang & Fan, 2011). Munne et al. (2010) conducted a research into dental placodes in mice. Dental placodes are epithelial structures that form on the dental lamina and develop into individual tooth germs (Jussila & Thesleff, 2012). They found that placode size is controlled by a balance between inhibitor and activator molecules, including BMPs and Activin. Disturbances to the reciprocal relationship between these signaling molecules can cause the placodes to split, creating additional teeth. The supernumerary tooth produced by this process is often supplemental in form but can be heteromorphic if the dental lamina does not split equally (Garvey et al., 1999; Liu, 1995). On occasion, the split may only be partial creating a double‐crowned gemmate tooth (Garattini et al., 1999).

Clinical studies on supernumerary teeth in Africa have revealed varying results. Several reports from Nigeria found that prevalence levels differed between the groups studied, ranging from 1.5–12.7%. Additionally, morphological differences and variations in the dental arch position most likely to be affected were observed (Adeyemi et al., 2012; Anibor et al., 2015; Bello et al., 2019; Ize‐Iyamu et al., 2016). A study of Sundanese students revealed a prevalence of 2.9%, with supplemental teeth the most common form (Abdulkareem & Abuaffan, 2016). The studies from Nigeria and Sudan showed no significant difference between males and females.

Supernumerary teeth have also been observed in historical and archaeological contexts around the world (Benazzi et al., 2010; Duncan, 2009; Sciulli, 1977; Suzuki et al., 1995), including South Africa (De Villiers, 1968; Randell, 1925; Shaw, 1931). Most recently, Van der Merwe and Steyn (2009) reported on a 19th century assemblage of migrant workers from South Africa with a relatively high incidence of supernumerary teeth at 6.7%, although only in the premolar and molar regions. Watters (1962) also found that 2.5–3% of several hundred indigenous West Africans had extra or supplemental teeth. Third premolars and fourth molars were fully formed and erupted. Again, no significant difference between the sexes was found. Additional cases of supernumerary molars have been reported in other archaeological collections from Africa (Irish, 2001; Rao, 1999).

Clinical data on gemination and fusion in Africa are sparse and focus on the primary dentition. Studies of children from Nigeria found differing prevalence rates ranging from 0.4–1.9% (Folayan et al., 2019; Onyeaso & Oneyeaso, 2006). Archaeological cases of double‐crowned teeth are extremely rare, with only a handful published to date, and most affected the primary dentition (Benazzi et al., 2010; Smith & Wojcinski, 2011; Tritsaroli, 2018). One historic archaeological example has been observed in sub‐Saharan Africa, that of an adult male with a double‐crowned tooth in a 19th century assemblage from Guinea (Irish, personal observation).

## 2 MATERIALS, METHODS, AND RESULTS

**FIGURE 1:**
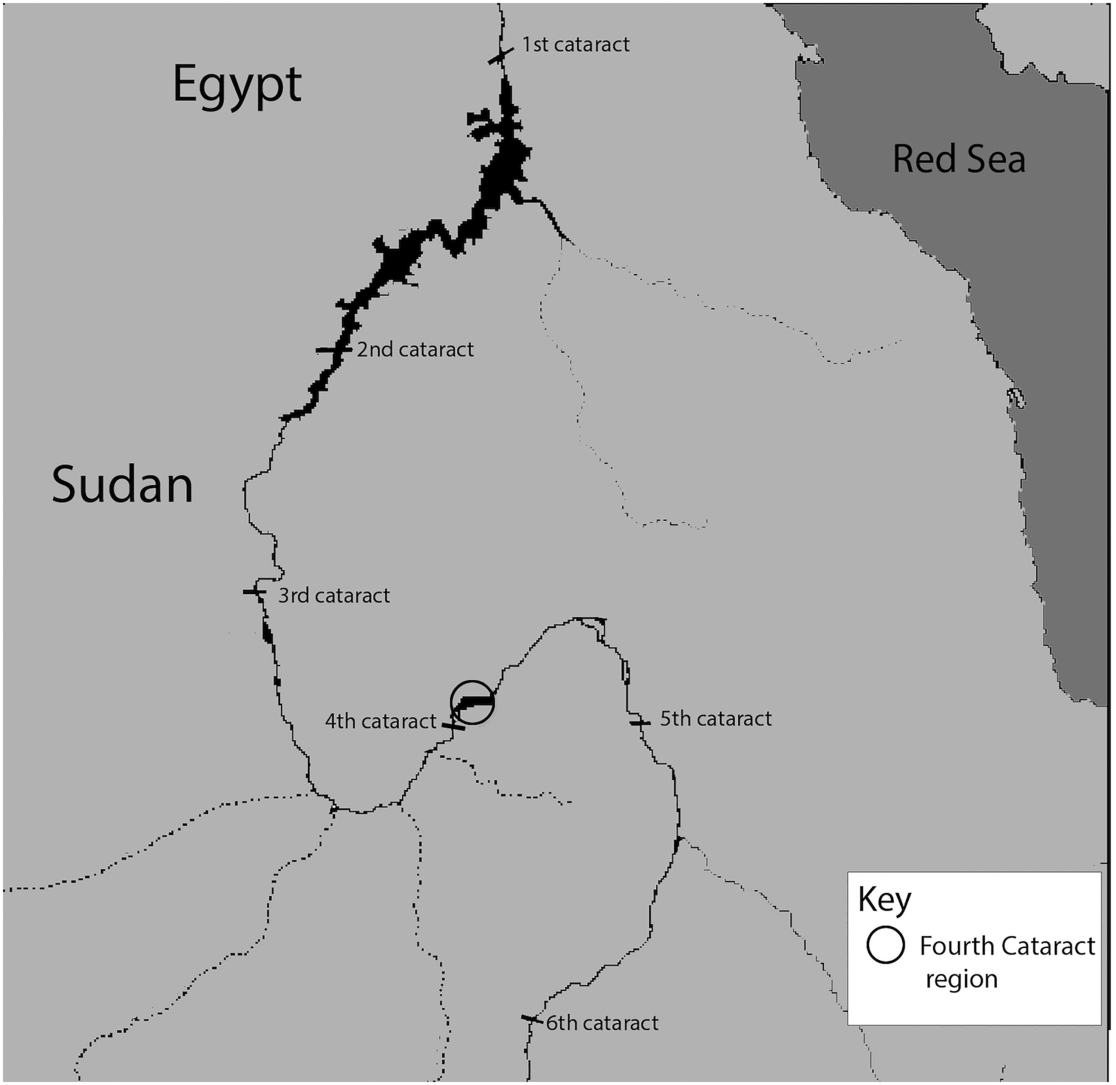
Map of ancient Nubia

During the analysis of an assemblage from the Fourth Cataract region of the Nile in northern Sudan (Figure 1), the remains of an individual with abnormally formed maxillary incisors was discovered. Dating to the medieval period [ca. AD 500–1500 in the region known as Nubia (Edwards, 2004)], the assemblage of 190 skeletons was excavated from cemetery 3‐J‐18, associated with a Christian church of the period (Ginns, 2010). The assemblage is part of the Fourth Cataract Collection, curated at the British Museum, which comprises 550 total skeletons from several sites excavated within a 20‐km‐wide area of the Nile Valley. The individual analyzed here was buried during the second phase of cemetery use, thought to be related to a time when the church was in active use. (Ginns, 2010). The body was buried in a supine position, orientated east–west. No grave goods were associated with the burial, but small pieces of textile were discovered at the base of the grave. The grave was marked by a rectangular mudbrick monument. These features are standard for this period in Nubia (Ginns, 2010).

**FIGURE 2:**
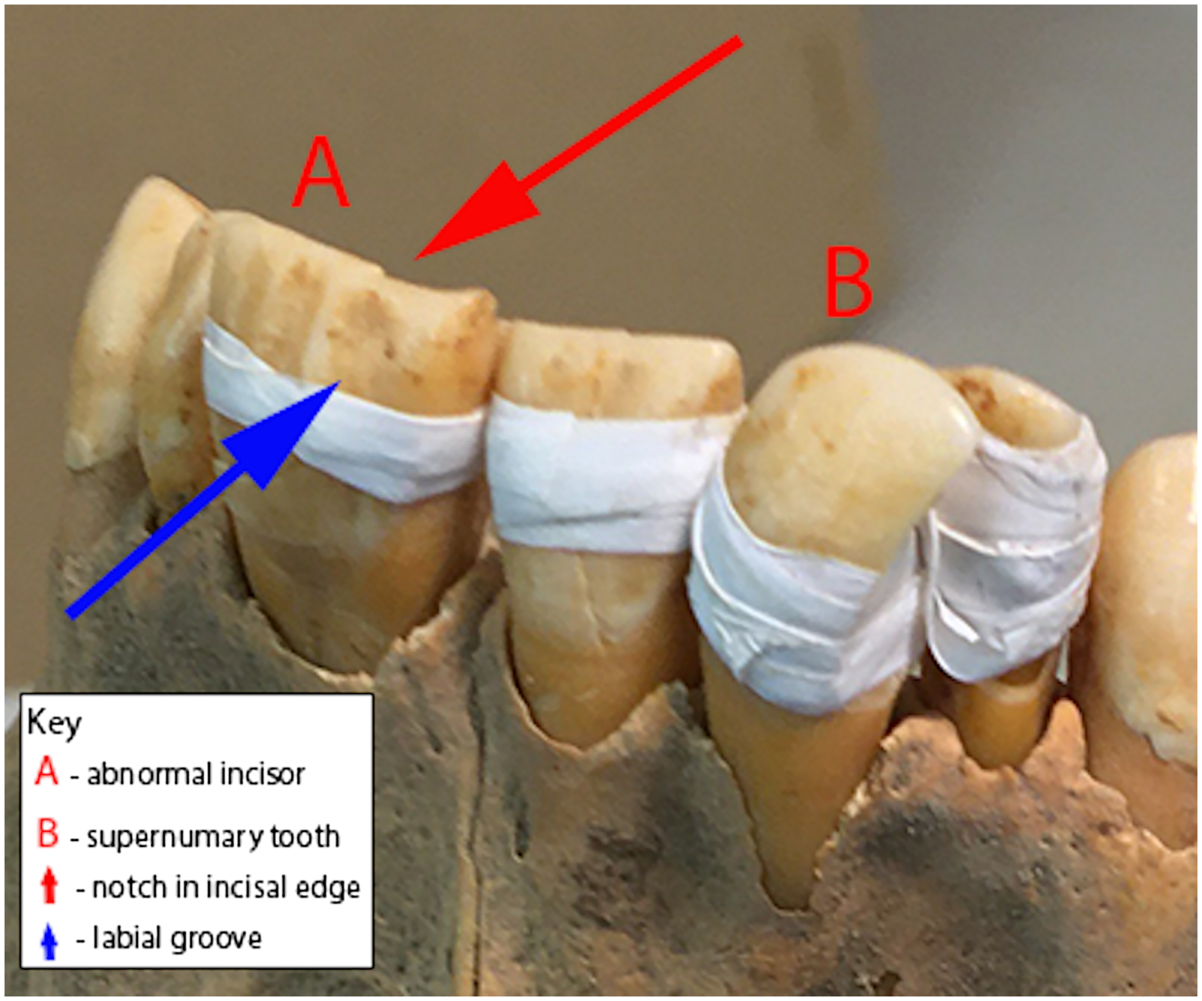
Labial view of anomalies [Colour figure can be viewed at wileyonlinelibrary.com]

**FIGURE 3:**
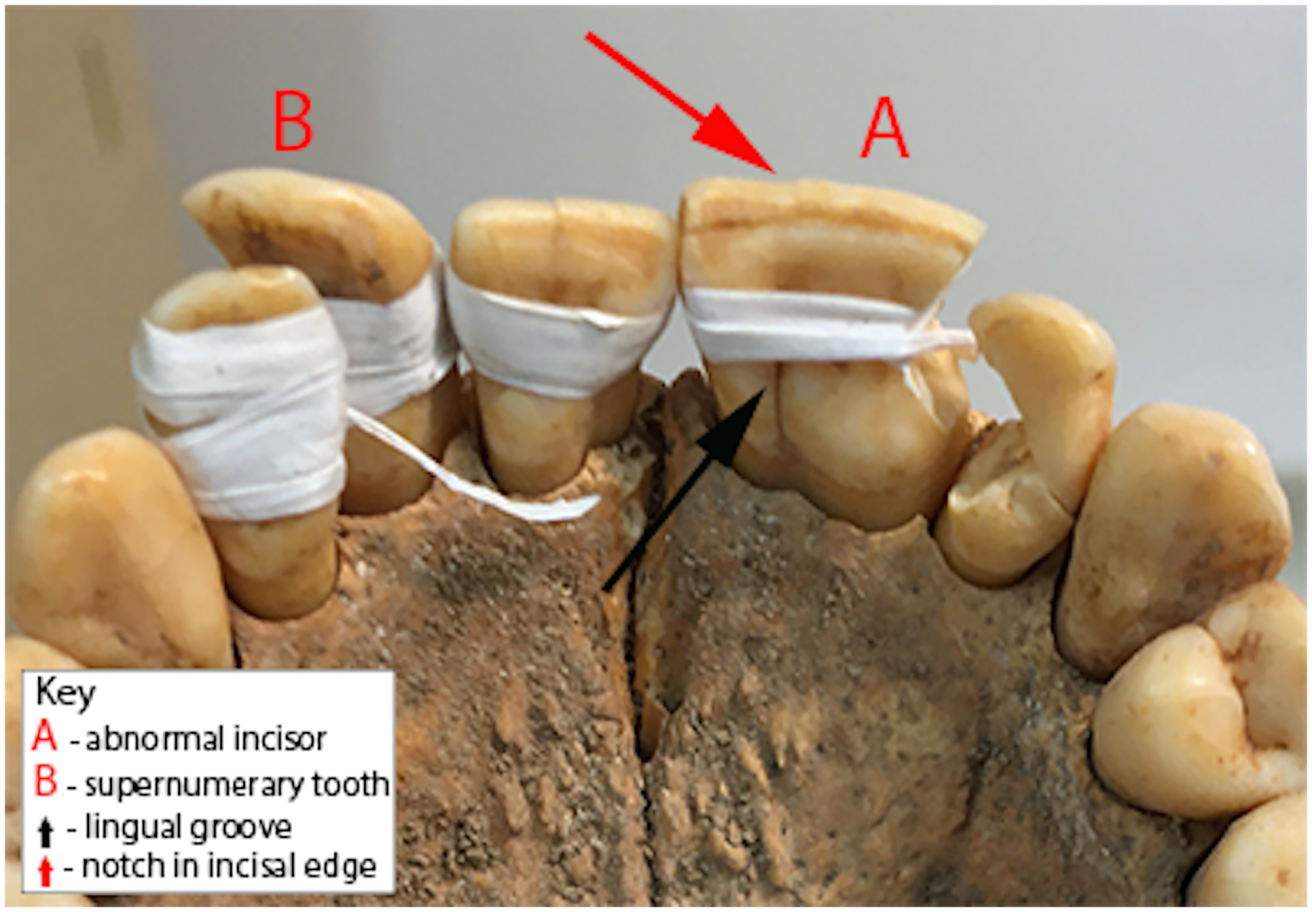
Lingual view of anomalies [Colour figure can be viewed at wileyonlinelibrary.com]

Using standard methods based on changes to pubic morphology, the individual was categorized as a young adult female aged 20–34 years (Bruzek, 2002; Buikstra & Ubelaker, 1994: 16–32). The skeleton is 95% complete. A partially healed fracture was recorded on the left second metacarpal, but no other pathological changes were observed in the skeleton or dentition. All permanent teeth were recovered in a good state of preservation, apart from the maxillary incisors that had been damaged post mortem. They were temporarily reconstructed and held together with static (PTFE™) tape—a British Museum passive conservation approach that is reversible, makes use of inert materials and avoids potentially damaging adhesives (see Wills & Antoine, 2015; Wills et al., 2014). The left central upper incisor has a double crown. A groove runs along the full length of the labial side, producing a small notch on the incisal edge of the crown (Figure 2). There is a corresponding groove on the lingual side (Figure 3). The root is wide, indicating the presence of two root canals (Figure 3). An extra tooth was also noted on the right side of the maxilla, between the central and lateral incisor (Figures 2 and 3). The latter is eumorphic in form, morphologically like the central incisor and of similar size. An additional socket is present on the right side of the maxilla. All other permanent teeth are present and normally formed.

Only one other supernumerary tooth was observed in the assemblage. A young male had a supernumerary incisor present on the left side of the maxilla. No further examples of a double‐crowned tooth were observed. The prevalence for supernumerary teeth in the assemblage is 1.72%, and 0.86% for double‐crowned teeth. The prevalence is based on the number of individuals with full permanent dentitions. If all 32 teeth had not been fully recovered, the relevant parts of the alveolar process needed to be present and observable for the individual to be included in the prevalence calculation.

## 3 DISCUSSION

The abnormal central incisors in this individual are rare in archaeological populations recorded to date. They represent the only case of a double crown yet found in ancient Nubians and one of the few supernumerary teeth. Fusion and gemination are seldom evident in the permanent dentition, and archaeological cases have only occasionally been reported. The present example adds to the documentation of supernumerary teeth in ancient times and, based on a review of anthropological literature, appears to be the earliest reported example of gemination/fusion in the permanent dentition.

Although two different dental anomalies are present, the bilateral expression suggests that the same biological mechanism could be responsible. If the abnormal odontogenesis of the left incisor had been caused by gemination, then the tooth germ may have attempted to split but failed to do so (Garattini et al., 1999). The latter process does not always fail and, on occasion, dichotomy of the tooth germ results. If dichotomy (full or partial) was the cause of both anomalies in the present individual, then there is asymmetry in expression. Whether a full split occurs is thought to depend on the developmental stage of the tooth (Grover & Lorton, 1985). If there was disparity in development between central incisors, it could have caused only a partial split on one side.

Another possible etiology for the abnormal upper left incisor is fusion. As the tooth count has not been affected, the central incisor must have fused with a supernumerary tooth. In such a situation, these teeth would have developed bilaterally. Fusion with supernumerary teeth has been observed in clinical and archaeological reports (Bennazi et al., 2010) and is often thought to occur when pressure or force result in contact between the developing tooth buds (Nunes et al., 2002). With two additional teeth in the dental arch in our example, the two tooth germs may have been forced to merge.

Recent research into the molecular mechanisms involved in tooth development has greatly advanced (Thesleff, 2006). Epigenetic processes and how they express the genome have been key in understanding the different stages of odontogenesis (Townsend et al., 2012). Both dichotomy and the occurrence of supernumerary teeth have been linked to the relationship between signaling molecules, which act as activators and inhibitors, and the dental lamina (Munne et al., 2010; Tummers & Thesleff, 2009). Without the interaction between dental lamina and these molecules, odontogenesis would not be possible (Wang & Fan, 2011). If the relationship between these two elements is disrupted, then abnormalities, like the ones reported here, can occur (Tummers & Thesleff, 2009).

Geographic‐ and population‐specific epidemiological patterning noted in the clinical literature for supernumerary teeth can also be observed in archaeological contexts. A higher propensity for supernumerary teeth in populations from specific geographical regions has been noted (Suzuki et al., 1995; Watters, 1962; Zhu et al., 1996). Furthermore, intraregional prevalence disparities have been reported using modern and historical data (Bello et al., 2019; Duncan, 2009). The prevalence rate for the Nubian site is 1.72%, which is within the prevalence range observed in clinical literature from worldwide populations (0.1–3.8%) (Takahashi et al., 2016), and also those reported in studies on modern Africans (1.5–12.7%) (Abdulkareem & Abuaffan, 2016; Adeyemi et al., 2012; Anibor et al., 2015; Bello et al., 2019; Ize‐Iyamu et al., 2016).

These data indicate a higher propensity for supernumerary teeth in African populations but also variation between groups. Studies from Nigeria have shown that prevalence rates can vary widely between populations that are geographically proximate, with a prevalence of 1.5% in Benin (Ize‐Iyamu et al., 2016) and 12.7% from Abraka (Anibor et al., 2015). Variation was also found in the type and positioning of the supernumerary teeth; some reported a propensity for additional teeth in the anterior regions of the maxilla (Ize‐Iyamu et al., 2016) or mandible (Anibor et al., 2015), while others observed more supernumerary teeth in the molar and premolar regions (Bello et al., 2019). This variation could be mirrored in the archaeological record. Apart from the individuals at site 3‐J‐18, no other cases of any supernumerary type was recorded in the Fourth Cataract Collection, which includes sites of similar age in a geographically delimited area. Additionally, published examples of such teeth from other African archaeological collections have been largely premolars and molars (Irish, 2001; Rao, 1999; Van der Merwe & Steyn, 2009), contrasting with the two instances of anterior teeth in this assemblage.

If fusion was the cause of the abnormal incisor then this would be an example of bilateral supernumerary teeth, which only happens in 12–23% of cases (Takahashi et al., 2016). Often more than one supernumerary tooth may be an indication that the individual had a genetic syndrome (Anthonappa et al., 2013). There is no evidence of pathological changes in this individual (except for the aforementioned fracture), so it is unlikely that the supernumerary teeth were syndromatic.

Double‐crowned teeth are extremely rare in the permanent dentition, only observed in 0.1–0.2% of populations worldwide (Koszowski et al., 2014). While the prevalence rate at this site (0.86%) is higher than that of in modern data, this abnormality is scarcely observed in the archaeological record. This case being one of only two known cases, both observed in African collections (Irish, personal observation). Conversely, examples of teeth with a twinned crown in the deciduous dentition have been observed in archaeological assemblages from several countries (Benazzi et al., 2010; Smith & Wojcinski, 2011; Tritsaroli, 2018). If the distribution of double‐crowned teeth in the permanent dentition mirrors modern clinical data, there may be other examples that remain unpublished or yet to be classified. Another reason for the paucity of cases in archaeological assemblages could be due to preservation issues or post mortem damage. Alternatively, permanent teeth with twinned crowns may have been less prevalent in non‐African past populations.

Additional studies of skeletal collections, both in Africa and worldwide are necessary to further understand the prevalence and expression of supernumerary teeth and gemination/fusion in the past and help contextualize this case study further.

This Nubian individual exemplifies what can occur when the epigenetic balance is disturbed. Publishing archaeological cases of these anomalies add to their history, allowing comparisons with modern data and furthering our understanding of how they were expressed in past populations.

## CONFLICT OF INTEREST

The authors declare no conflict of interest.

## Data Availability

The data that support the findings of this study are available from the corresponding author, E. L. W. P, upon reasonable request.
